# Confounding Roles of ER Stress and the Unfolded Protein Response in Skeletal Muscle Atrophy

**DOI:** 10.3390/ijms22052567

**Published:** 2021-03-04

**Authors:** Yann S. Gallot, Kyle R. Bohnert

**Affiliations:** 1 LBEPS, Univ Evry, IRBA, Université Paris Saclay, 91025 Evry, France; 2 Kinesiology Department, St. Ambrose University, Davenport, IA 52803, USA

**Keywords:** skeletal muscle, atrophy, muscle wasting, ER stress, UPR

## Abstract

Skeletal muscle is an essential organ, responsible for many physiological functions such as breathing, locomotion, postural maintenance, thermoregulation, and metabolism. Interestingly, skeletal muscle is a highly plastic tissue, capable of adapting to anabolic and catabolic stimuli. Skeletal muscle contains a specialized smooth endoplasmic reticulum (ER), known as the sarcoplasmic reticulum, composed of an extensive network of tubules. In addition to the role of folding and trafficking proteins within the cell, this specialized organelle is responsible for the regulated release of calcium ions (Ca^2+^) into the cytoplasm to trigger a muscle contraction. Under various stimuli, such as exercise, hypoxia, imbalances in calcium levels, ER homeostasis is disturbed and the amount of misfolded and/or unfolded proteins accumulates in the ER. This accumulation of misfolded/unfolded protein causes ER stress and leads to the activation of the unfolded protein response (UPR). Interestingly, the role of the UPR in skeletal muscle has only just begun to be elucidated. Accumulating evidence suggests that ER stress and UPR markers are drastically induced in various catabolic stimuli including cachexia, denervation, nutrient deprivation, aging, and disease. Evidence indicates some of these molecules appear to be aiding the skeletal muscle in regaining homeostasis whereas others demonstrate the ability to drive the atrophy. Continued investigations into the individual molecules of this complex pathway are necessary to fully understand the mechanisms.

## 1. Introduction

Skeletal muscle, which accounts for 30 to 40% of human body weight [1,2] is an essential organ, responsible for many physiological functions such as breathing, locomotion, postural maintenance, thermoregulation, and metabolism. This tissue constitutes an important reservoir of proteins, especially myofibrillar proteins, since these proteins represent 75 to 80% of the volume of the muscle fiber [3]. Additionally, skeletal muscle is a dynamic and plastic tissue, capable of adapting and changing to external stressors. To maintain plasticity, skeletal muscle utilizes multiple pathways that regulate cell and protein content [4]. The loss of skeletal muscle mass, commonly referred to as muscle atrophy or wasting, is associated with various physiological and pathological conditions such as aging, trauma, immobilization, denervation, cancer, diabetes, inflammation, and starvation [5]. Additionally, genetic muscle disorders or myopathies are also characterized by the continual loss of skeletal muscle. This process of muscle loss is combated by continual regeneration of the muscle until ultimately fibrosis occurs and eventual failure of the respiratory muscles [6]. Thus, the development of treatments to slow down or prevent the loss of skeletal muscle is essential to counteract numerous pathologies. However, the complete understanding of the molecular mechanisms of skeletal muscle atrophy needed for such therapeutics remains enigmatic.

**Figure 1:**
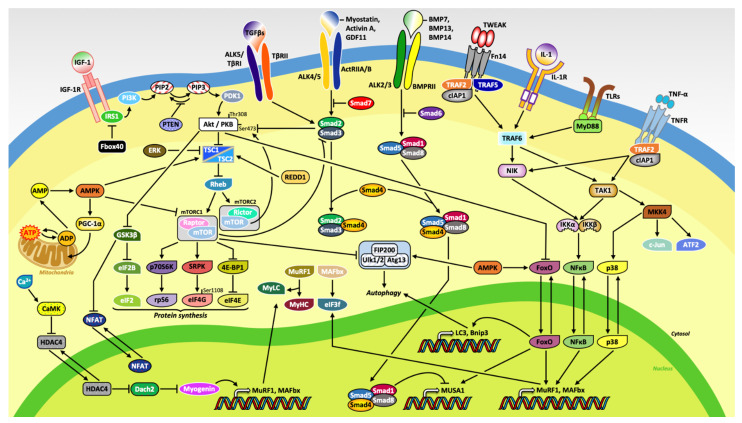
Schematic representation of the molecular mechanisms regulating skeletal muscle mass. Binding of insulin-like growth factor-1 (IGF-1) to its putative receptor (IGF-1R) induces protein synthesis primarily through downstream activation of Akt/PKB and mTOR. The myostatin/activin A/growth differentiation factor 11 (GDF11) and transforming growth factor beta (TGFβ) pathways inhibits muscle growth due to the phosphorylation of Smad2/3, which serves a primary function as an inhibitor of Akt. The ubiquitous protein kinase complex mammalian target of rapamycin complex 1 (mTORC1) stimulates protein synthesis through activation of the 70-kDa ribosomal protein S6 kinase (p70S6K) and its substrate ribosomal protein S6 (rpS6), the serine/arginine-rich protein specific kinase SRPK, and the translation repressor protein called eukaryotic translation initiation factor 4E (eIF4E)-binding protein 1 (4E-BP1), also known as PHAS-1. Moreover, increased in cellular concentration of adenosine monophosphate (AMP) stimulates AMP-activated protein kinase (AMPK) as well as peroxisome proliferator–activated receptor gamma coactivator-1 alpha (PGC-1α) to induce mitochondrial biogenesis. Extracellular signaling molecules, bone morphogenic protein (BMP) 7, 13, and 14, and inflammatory cytokines tumor necrosis factor (TNF)-like weak inducer of apoptosis (TWEAK), interleukins (ILs), and tumor necrosis factor alpha (TNF-α) increased expression of genes related to the ubiquitin-proteasome system (UPS) and autophagic-lysosomal pathway (ALP).

Skeletal muscle atrophy leads to muscle weakness resulting in not only a diminished quality of life but also in an increase in both morbidity and mortality in numerous pathologies [4]. Skeletal muscle mass is determined by the delicate equilibrium between rates of protein synthesis and degradation (Figure 1). The ubiquitin-proteasome system (UPS) represents one of the major proteolytic mechanism in skeletal muscle [7,8,9]. In addition to the well-known muscle-specific E3 ubiquitin ligases, muscle RING finger 1 (MuRF1 also known as TRIM63; [10]) and muscle atrophy F-box (MAFbx)/atrogin-1, several other key ligases such as neural precursor cell expressed developmentally down-regulated protein 4.1 (Nedd4.1), tumor necrosis factor receptor (TNFR)-associated factor 6 (TRAF6), and muscle ubiquitin ligase of the SCF complex in atrophy-1 (MUSA1), have been identified and mediate proteolytic degradation of both thick and thin filaments in skeletal muscle [4,11,12]. The autophagic-lysosomal pathway (ALP) is another preeminent catabolic mechanism involved in the clearance of cytoplasmic components such as defunct organelles as well as misfolded or other harmful proteins [13,14]. Although research has shown that suppression of basal autophagy can be detrimental and lead to myopathies due to the excessive amounts of dysfunctional organelles as well as the initiation of oxidative stress [15,16], excessive activation of the ALP can also provoke muscle wasting [4,17,18,19]. An abundance of evidence has shown that the activity of the UPS and ALP are significantly influenced by the activation of a number of signaling pathways such as p38 mitogen-activated protein kinase (MAPK), adenosine monophosphate (AMP)-activated protein kinase (AMPK), and nuclear factor-kappa B (NF-κB). These pathways function through modulating the activity of various transcriptional regulators [4,5]. The insulin-like growth factor-1 (IGF-1)/phosphatidylinositol 3-kinase (PI3K)/protein kinase B (Akt)/mammalian target of rapamycin (mTOR) signaling axis is a critical intracellular signaling pathway that stimulates myofibrillar protein synthesis rates [9,14,20]. In addition, activation of the mTOR pathway inhibits the UPS and ALP in skeletal muscle [4,9,20]. Moreover, many studies report that changes in mitochondrial content and function also play a pivotal role in the regulation of skeletal muscle mass [4,21].

Skeletal muscle contains a specialized endoplasmic reticulum (ER), known as the sarcoplasmic reticulum (a type of smooth ER in myocytes), composed of an extended network of tubules. This membrane-bound organelle is responsible for the regulated release of calcium ions (Ca^2+^) into the cytoplasm to trigger muscle contraction [22]. In addition to its notable role in calcium homeostasis, this large and dynamic structure is also responsible for the proper folding, packaging, and trafficking of freshly synthesized secretory and transmembrane proteins. While protein processing in the ER normally occurs in an orderly fashion, a number of physiological and pathological stimuli, such as exercise, hypoxia, imbalances in calcium level, alteration in the redox balance, nutrient/energy deprivation, or even viral/bacterial infections can disturb ER homeostasis [23,24,25,26,27,28]. This dysfunction causes an accumulation of unfolded and misfolded proteins in the ER lumen, which may affect cellular function and create a toxic environment in the cell, leading to its death. In order to cope with this process known as ER stress, eukaryotic cells elicit a conserved adaptive mechanism, the unfolded protein response (UPR), aiming to increase production of ER chaperones, to clear damaged proteins, and to re-establish ER homeostasis [29]. The UPR is initiated by three ER transmembrane sensors: activating transcription factor-6 (ATF6), inositol-requiring protein (IRE) 1α, and protein kinase R (PKR)-like endoplasmic reticulum kinase (PERK) [23,28,30,31,32].

**Figure 2:**
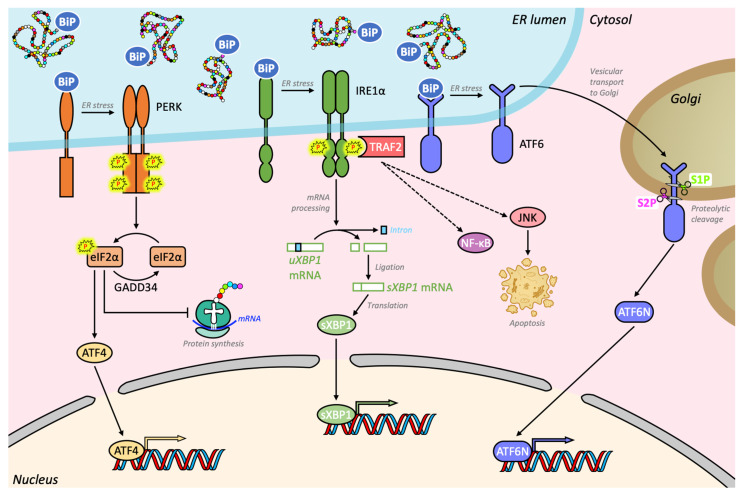
Schematic representation of the unfolded protein response (UPR) pathways in skeletal muscle. The three transmembrane proteins, PERK, IRE1α, and ATF6, are inactive when bound by BiP, also called GRP78. Due to the accumulation of misfolded proteins, GRP78/BiP disassociate from these proteins and preferentially binds to these proteins in the lumen of the ER. No longer inactivated by GRP78/BiP, PERK auto-phosphorylates causing phosphorylation of the translation initiation factor eIF2α and translation of the mRNA encoding ATF4, a potent transcription factor, which in turn can activate its transcriptional targets. One of these targets encodes GADD34, a phosphatase involved in the negative feedback control of this pathway, which allows a return to a physiological translational state. Another ER transmembrane sensor, IRE1α, is also activated by autophosphorylation during ER stress. When active, IRE1α catalyzes the unconventional splicing of a 26-base intron from XBP1 mRNA through its endoribonuclease activity, which generates the highly active transcription factor transcription factor sXBP1, as well as phosphorylates c-Jun N-terminal kinase (JNK). Lastly, once disassociated from GRP78/BiP, the membrane-bound ATF6 is released and traffics from the ER to the Golgi apparatus where it undergoes two sequential proteolytic cleavages, mediated by the site-1 and site-2 proteases (S1P and S2P). The cleaved cytosolic N-terminal fragment of ATF6 (ATF6N) translocates to the nucleus where it acts in combination with sXBP1 and ATF4 to alleviate ER stress by regulating gene expression and protein synthesis.

In a naïve state, the three transmembrane proteins (ATF6, IRE1α, PERK) are held in an inactive state by binding to the ER resident chaperone 78 kDa glucose-regulated protein (GRP78), also known as binding immunoglobulin protein (BiP). During stress to the ER, GRP78/BiP disassociates from these proteins to preferentially bind to the misfolded/unfolded proteins in the ER lumen (Figure 2). Once GRP78/BiP is released, PERK (also known as EIF2AK3) is auto-phosphorylated leading to a cascade of signals including direct phosphorylation of eukaryotic translation initiation factor 2α (eIF2α) and translation of activating transcription factor-4 (ATF4), which leads to the induction of a pro-apoptotic transcription factor called CCAAT enhancer binding protein (C/EBP) homologous protein (CHOP) [23,28,33,34]. Additionally, the PERK/eIF2α axis has a role in the termination of the UPR by the induction of growth arrest and DNA damage-inducible protein 34 (GADD34) [35]. Another ER transmembrane sensor, IRE1α (also known as ERN1), also becomes activated by autophosphorylation during ER stress. Through its endonuclease activity, IRE1α promotes splicing of a 26-base intron from X-box-binding protein 1 (XBP1) mRNA [32,36]. Spliced XBP1 (sXBP1) is an active transcription factor and induces the gene expression of ER chaperones and other components of the UPR in order to boost the folding capacity of the ER [37]. Lastly, once activated, ATF6 (also known as ATF6α) is transported from the ER to the Golgi apparatus where it is cleaved by site-1 protease (S1P) and site-2 protease (S2P) [38,39,40]. Similar to sXBP1, the cleaved N-terminal fragment of ATF6 (ATF6N) is also an active transcription factor that is trafficked to the nucleus to aide with increasing the genetic expression of UPR proteins to suppress ER stress [36]. The induction of UPR genes stimulates the protein folding capacity of the cell leading to a decrease in the misfolded/unfolded protein load [23,28,33,34]. However, over-activation of the UPR can induce apoptosis of the cell through each of the three pathways.

While chronic, unmitigated ER stress can lead to cell death, accumulating evidence suggests that a basal levels of ER stress may protect the cell from future perturbations, a process named ER hormesis. Indeed, the UPR has been found to be essential for regulation of autophagy, mitochondrial biogenesis and function, and the expression of various antioxidant molecules to protect mammalian cells under stressed conditions [41]. Moreover, basal levels of ER stress and the UPR also play aid in stem cell function and survival in various organs [42,43]. Moreover, various tissues have indicated that each arm of the UPR may have a distinct role in regulation of cell fate in both naïve and diseased states [23,28,30,31].

Recently, ER stress and the UPR has been the focus of numerous investigations attempting to understand the role of the UPR in skeletal muscle homeostasis and disease. In the following sections, we provide a succinct review of the role of ER stress-induced UPR pathways in the regulation of skeletal muscle atrophy in various different conditions, including: cancer cachexia, denervation, disuse, starvation, sarcopenia, and myopathies as well as during skeletal muscle loading.

## 2. Cancer Cachexia

Cancer cachexia is a devastating syndrome that is characterized by the progressive and uncontrolled loss of body weight that results from depletion of skeletal muscle and adipose tissue [44]. Cachexia afflicts a wide array of tumors and is estimated to be responsible for about 20–30% of cancer-related deaths [45,46]. Additionally, cancer cachexia leads to intolerance of chemotherapies and other antineoplastic treatments and decreases survival of patients with cancer [47,48,49,50]. Recent studies have indicated that the loss of muscle mass due to cachexia is caused by excess activation of both the UPS and ALP. However, the upstream mechanisms activating these pathways in skeletal muscle has yet to be fully elucidated.

The UPR has been demonstrated to be essential in the regulation of skeletal muscle mass during cancer cachexia. Specifically, recent studies have reported that several markers of ER stress and the UPR are induced in skeletal muscle of two models of cancer cachexia (Lewis lung carcinoma (LLC)-bearing and Apc^Min/+^) [51]. Moreover, LLC conditioned medium was sufficient to induce the phosphorylation of eIF2α, splicing of XBP1, and expression of several downstream UPR markers. These results indicate that instead of a systemic effect, factors derived from the tumors are responsible for the activation of the UPR in skeletal muscle. Moreover, pan-inhibition of ER stress using 4-phenylbutyrate (4-PBA), a chemical chaperone known to attenuate ER stress both in vivo and in vitro [52,53], exacerbated the muscle loss in LLC-bearing mice [51]. Interestingly, chronic administration of 4-PBA induced atrophy in naïve skeletal muscle as well as primary myotubes. Results from this study indicated that treatment of 4-PBA reduced the activity of the Akt/mTOR pathway and increased the expression of components of both the UPS and the ALP in LLC-bearing mice [51]. However, one limitation of this study was that the prolonged use of 4-PBA can have other systemic effects in addition to its effect on the ER stress pathway [51]. More recently, a study determined that models of ovarian cancer were also sufficient to induce ER stress, specifically the PERK arm [54]. However, when treated with the steroidal lactone Withaferin A (WFA), a therapeutic agent known to attenuate cachexia [55], the mice preferentially expressed the IRE1 arm of the UPR. These data further indicate the possibility of deferential effects of different component of the UPR during cancer cachexia. Therefore, in order to fully understand the role of the UPR and ER stress in cancer cachexia, genetic mouse models were necessary.

In recent studies, researchers used genetic mouse models to investigate both the PERK and IRE1α arm of the UPR in cancer cachexia. These results demonstrated that the targeted inducible deletion of PERK reduces morphological and functional outcomes of skeletal muscle [56]. Furthermore, short hairpin RNA (shRNA)-mediated knockdown or pharmacologic inhibition of PERK caused atrophy in cultured myotubes. In addition to increasing the rate of protein synthesis, genetic deletion of PERK led to the increased expression of components of the UPS and ALP in skeletal muscle. Moreover, ablation of PERK also increased the activation of intracellular Ca^2+^-dependent cysteine proteases called calpains and deregulated the gene expression of the members of the fibroblast growth factor (FGF) 19 subfamily. Interestingly, the targeted deletion of PERK exacerbated the skeletal muscle wasting in LLC-bearing mice by causing elevated levels of both the UPS and ALP. Interestingly, deletion of PERK significantly inhibited the levels of ATF4 in cachectic mice. ATF4 is a basic-leucine zipper transcription factor that is induced through the activation of the PERK arm of the UPR. Previous studies have indicated that activation of ATF4 mediates muscle wasting during aging [57], and also during starvation [58] through activation of growth arrest and DNA damage-inducible protein 45 alpha (GADD45a) [59], an 18-kDa globular protein shown to promote skeletal muscle atrophy [60,61]. Moreover, we found increased LLC-bearing mice also express ATF4 at increased levels and those levels were abolished by the administration of 4-PBA [51]. However, in both PERK knockdown of 4-PBA treated mice we still observed accelerated muscle atrophy. These findings suggest that ATF4 may not be involved in muscle wasting during cancer cachexia. Nevertheless, the study provided initial evidence that the activity of the PERK arm is essential for maintaining muscle mass in naïve conditions and during cancer cachexia.

Muscle-specific deletion of XBP1, a major downstream target of IRE1α arm of the UPR, ameliorates muscle wasting in LLC-bearing mice [62]. Our results also demonstrate that overexpression of the spliced form of XBP1 caused atrophy in cultured myotubes. In contrast, knockdown of XBP1 specifically in skeletal muscle stunted myotube atrophy in response to both LLC and colon-26 (C26) adenocarcinoma cell conditioned medium. Interestingly, this study demonstrated that this arm of the UPR is controlled by the activation of the Toll-like receptor (TLR)/myeloid differentiation primary response gene 88 (MyD88) signaling axis.

Well described in cancer patients, cachexia also occurs in many other chronic conditions such as chronic heart failure (CHF), cystic fibrosis, rheumatoid arthritis, acquired immune deficiency syndrome (AIDS), chronic obstructive pulmonary disease (COPD), and chronic kidney disease (CKD). CKD-associated cachexia shares common features with the cancer-associated cachexia, in particular, a progressive loss of body weight and adipose tissue as well as a marked muscle wasting [63,64]. In a recent study, researchers showed that ER stress is involved in muscle atrophy in CKD [65]. Indeed, using adenine-induced CKD model rodents, they highlighted a decrease in muscle mass associated with an increased mRNA expression of certain ER stress markers (GRP78/BiP, sXBP1 and ATF4 in rats, and GRP78/BiP, sXBP1, ATF4 and CHOP in mice). The investigators also reported that the injection of the chemical chaperone 4-PBA in mice improved muscle atrophy and significantly reduced the levels of gene expression of UPR.

Taken together, these results provide clear evidence that ER stress and the UPR pathways are activated in numerous different models of cancer cachexia, and in CKD-associated cachexia. Although pan-inhibition of the UPR using 4-PBA may cause deleterious effect, it appears different arms of the UPR may have roles in cancer cachexia. Further elucidation of individual arms and molecules is necessary to tease out the differing mechanisms of these pathways in cancer cachexia.

## 3. Denervation

Skeletal muscles contain connective tissue and possess a profuse supply of blood vessels and nerves. Innervation has a pivotal role and is particularly required during the developmental process of skeletal muscle and for the maintenance its postnatal growth [66,67]. In the absence of innervation or following the removal of nerves, skeletal muscle is progressively lost, leading to a robust skeletal muscle atrophy with a marked decrease in contractile force production [68]. Denervation is generally associated with neoplasia, cancer surgery, trauma, viral infections, neuromuscular diseases including spinal muscular atrophy (SMA), amyotrophic lateral sclerosis (ALS), and peripheral neuropathies such as Charcot-Marie-Tooth disease (CMT). The effect of denervation on skeletal muscle can be detrimental, leading to weakness, loss of motor function, respiratory failure, and eventually mortality [69,70,71,72]. However, despite the prevalence and severity of denervation-induced muscle atrophy, the molecular mechanisms that regulate the loss of muscle mass has yet to be determined.

Several markers of ER stress and the UPR, including CHOP, are increased in skeletal muscle in response to denervation and in a mouse model of spinal and bulbar muscular atrophy (SBMA) generated through gene targeting (AR113Q mice) [73]. Interestingly, genetic deletion of CHOP accentuated muscle atrophy in mice after denervation and in the AR113Q mice. Interestingly, the increase in muscle loss was not due to a noticeable difference in the activity of the UPS. Instead, the deletion of CHOP stimulated the ALP in the skeletal muscle of both of these models. Moreover, Beclin-1 haploinsufficiency causing attenuation of autophagy stunted muscle atrophy in response to denervation in naïve mice and increased the lifespan of AR113Q mice [73]. Although the mechanisms by which CHOP inhibits autophagy remain unknown, these findings suggest that induction of CHOP could be a mechanism to prevent excessive loss of muscle mass in response to denervation and during SBMA.

ER stress and the UPR pathways are also activated in the Cu/Zn superoxide dismutase (SOD1) models of ALS [74,75]. Specifically, the PERK and IRE1α arm of the UPR were activated in early, presymptomatic stages, and continued to increase as the disease progressed [74]. Interestingly, induction of the ALP following XBP1 deletion was shown to ameliorate the pathology of SOD1 mice. In this model, XBP1 deficiency caused increased expression of the ALP which promoted the degradation of mutant SOD1 resulting in the clearance of aggregates [75]. These reports indicate that XBP1 may have deferential mechanisms depending on the underlying condition and muscle wasting stimuli. Therefore, additional investigations of genetic mouse models of the UPR are essential to continue to understand the role in denervation induced atrophy and in neuromuscular diseases.

## 4. Disuse/Unloading

Injuries and illnesses typically involve a period of disuse or unloading. The use of unloading can have therapeutic effects allowing healing of a broken bone or injured muscle. However, the unloading leads to disuse of the skeletal muscle leading to skeletal muscle atrophy. The loss of muscle mass due to disuse can delay recovery and attempts to prevent the atrophy through physical therapy or other therapies has been insufficient [76,77,78]. Although disuse-induced skeletal muscle atrophy is a common and serious problem in wide array of medical situations, it is not well understood at the molecular level.

An earlier study reported no significant changes in the expression of UPR markers such as GRP78/BiP, calreticulin, CHOP, the cytoskeletal protein vinculin, the type I D-myo-inositol 1,4,5-trisphosphate receptor (IP_3_R), protein kinase R (PKR), and eIF2α in skeletal muscle in response to inactivity [79]. Interestingly, both GRP78/BiP and CHOP were also shown to be increased 14 days after reloading the muscle, an effect that was more pronounced in elderly mice [80]. Additionally, ATF4, downstream transcription factor of the PERK, is also elevated in skeletal muscle after inactivity [81]. Furthermore, mice having ATF4 knocked down in muscle were partially resistant to inactivity-induced muscle wasting and forced expression ATF4 induced muscle fiber atrophy in the absence of inactivity. Further investigation of all UPR pathways is necessary to understand the role of ER stress in disuse-induced skeletal muscle atrophy.

## 5. Starvation

Fasting/nutritional deprivation can lead to a robust loss of skeletal muscle tissue. This skeletal muscle wasting has many similar mechanisms of other atrophic stimuli [82]. Similar to other wasting conditions, the loss of muscle mass upon starvation involves activation of both the UPS and the ALP. However, a distinct mechanism of starvation induced atrophy is that muscle proteins are degraded and mobilized for amino acid production, which is further used for gluconeogenesis. Moreover, additional evidence indicates other distinct qualities of starvation-induced atrophy, such as altered levels of insulin growth factors and glucocorticoids [83,84]. Therefore, work to understand the intricacies of skeletal muscle wasting due to starvation continues to be investigated.

Recently, ATF4, downstream transcription factor of the PERK arm, was found to be involved in starvation-induced of skeletal muscle atrophy. Specifically, fasting increases the level of ATF4 mRNA. Inhibition of ATF4 expression with an RNA targeting ATF4 and a phosphorylation-resistant form of eIF2α reduced myofiber atrophy during fasting. Moreover, overexpressed ATF4 reduced myofiber size in the absence of fasting. Interestingly, a transcriptionally inactive ATF4 construct did not reduce myofiber size, suggesting a requirement for ATF4-mediated transcriptional regulation [85].

TRAF6 is an E3 ubiquitin ligase, responsible for tagging proteins with ubiquitin to be degraded by the 20S proteasome. TRAF6 functions as a central regulator in multiple signaling pathways, such as NF-κB, MAPK, and PI3K/Akt, in response to certain cytokines and microbial products. Skeletal muscle specific deletion of TRAF6 inhibits skeletal muscle wasting in multiple distinct catabolic conditions including starvation, denervation, and cancer cachexia [58,86]. Interestingly, TRAF6 appears to be a potential upstream regulator of the UPR in skeletal muscle, especially in response to starvation. Specifically, studies have shown that deletion of TRAF6 in skeletal muscle reduces the levels of multiple markers of the UPR such as ATF4, CHOP, the specific heat shock protein 90 (HSP90) family member glucose-regulated protein 94 (GRP94), and GADD34 in fasted mice [58]. However, the mechanism by which TRAF6 and the UPR are connected in skeletal muscle during starvation remain to be investigated. More investigations are needed to fully elucidate all how all three arms of the UPR interact to mediate skeletal muscle atrophy due to nutrient deprivation.

## 6. Sarcopenia

The progressive loss of muscle mass and function with age is referred to as sarcopenia. Although the loss of muscle mass due to age seems unavoidable, it is critical for elderly individuals to maintain a high level of mobility [87,88]. The evidence is clear that the cause of sarcopenia is multifactorial [89,90,91,92]. At cellular and molecular levels, one potential factor of sarcopenia is ER stress. Specifically, ER stress was found to be elevated in the skeletal muscle of both elderly human and rodent [93,94,95,96]. Additionally, activation of ER stress has been implicated in diaphragm contractile dysfunction in a mouse model of sepsis [97]. An established mechanism in sarcopenia is anabolic resistance, which is defined as the reduced ability of skeletal muscle to increase protein synthesis in response to amino acids and protein, insulin, or exercise. However, ER stress was recently shown to not contribute to age-associated anabolic resistance in the skeletal muscle of mice [93].

Another factor of sarcopenia is mitochondrial dysfunction. Sedentary individuals display an age-related decline in the mitochondrial protein, optic atrophy 1 (OPA1), which is associated with muscle loss. Genetic deletion of Opa1 induced a precocious senescence phenotype and premature death [98]. Moreover, the ablation of Opa1 leads to ER stress inducing a catabolic program of muscle loss and systemic aging. Furthermore, deletion of both Opa1 and dynamin-related protein 1 (DRP1) further exacerbated the ageing phenotype and ER stress induction [99]. Moreover, removal of just DRP1 is also sufficient to induce skeletal muscle atrophy as well as ER stress [100]. Interestingly, pharmacological inhibition of ER stress by tauroursodeoxycholic acid (TUDCA) compensates for the loss of Opa1 and prevented muscle atrophy and premature death. These results indicate that mitochondrial dysfunction can trigger a cascade of signaling initiated at the ER that systemically affects general metabolism and aging.

## 7. Myopathy

Myopathies are muscle diseases in which the muscle fibers experience inflammation and as a result are damaged [101]. More specifically, myositis is a myopathy in which the inflammation of skeletal muscle leads to function deficits, such as weakness, swelling, and pain. However, several studies have indicated that inflammation alone does not cause myositis, indicating additional potential causes [102]. A common model for myositis is to overexpress class I major histocompatibility complex (MHC-I) in skeletal muscle using a conditional transgenic mouse. Interestingly, these transgenic mice display increased ER stress, as indicated by the expression of GRP78/BiP [103]. Moreover, when the immunodeficient Rag2^−/−^ mouse was forced to express MHC-I, the prevention of the immune response still caused myositis to occur as well as an increase in the UPR genes [104].

Sporadic inclusion body myositis (sIBM) is the most common degenerative muscle disease in adults over the age of 50. This disease is known to induce vacuolar muscle fibers, intramuscular fiber inclusions, and various degrees of mononuclear cell inflammation [105,106]. Interestingly, similar to Alzheimer’s disease, sIBM is characterized by accumulation of amyloid-β (Aβ) and phosphorylated tau proteins [105,106]. Recent studies in human’s have shown that the expression of all major ER chaperones is increased, and they associate with the Aβ proteins in skeletal muscle biopsies from patients with sIBM [107]. Possibly, ER chaperone proteins could be essential for the proper folding and trafficking of Aβ in skeletal muscle. Moreover, muscle biopsies of sIBM patients have also shown increases in other UPR genes including ATF4, CHOP, ATF6, and XBP1 [108].

Myasthenia gravis (MG) is an autoimmune disorder that is characterized by pathogenic autoantibodies that attack acetylcholine receptors (AChR) affecting the neuromuscular junction. Specifically, these autoantibodies impair the postsynaptic membrane by inducing complement-mediated damage, blocking the interaction of acetylcholine to the AChR causing degradation of AChR [109]. These mechanisms causing patients with MG to functionally experience weakness and fatigability. Interestingly, skeletal muscle biopsies from patients with MG exhibited an increase in GRP78/BiP and GRP94 [110,111]. Moreover, C2C12 myotubes that were treated with tunicamycin, an antibiotic commonly known to induce ER stress, exhibited increased degradation of the AChRs by endocytosis. Furthermore, silencing of XBP1, a downstream target of IRE1α, using small interfering RNA (siRNA) in C2C12 myotubes slowed the degradation of AChR by endocytosis [112].

Duchenne muscular dystrophy (DMD) is the most common muscular dystrophy and is characterized by a loss of the dystrophin protein leading to inflammation, cell infiltration, fibrosis, and necrosis of skeletal muscle [113]. The X-linked muscular dystrophy (mdx) mouse is the most widely used animal model for the DMD. Analysis of skeletal muscle from multiple studies in dystrophic mice showed an increase in numerous ER stress markers including GRP78/BiP, PERK, eIF2α, IRE1α, and sXBP1 [114,115]. Another class of muscular dystrophy is myotonic dystrophy type 1 (DM1). DM1 is an inherited muscular dystrophy which is caused by an abnormal amount of CTG trinucleotide repeats in the 3′ untranslated region of the dystrophia myotonica protein kinase (DMPK) gene. Skeletal muscle biopsies from patients with DM1 indicate increases in ER stress markers such as GRP78/BiP, PERK, eIF2α, and XBP1 [116]. Finally, oculopharyngeal muscular dystrophy (OPMD) is a rare late onset genetic disease leading to ptosis, dysphagia and proximal limb muscles at later stages [117]. A short abnormal (GCN) triplet expansion in the polyA-binding protein nuclear 1 (PABPN1) gene leads to PABPN1-containing aggregates in the muscles of OPMD patients. Recently, treatment of mice with guanabenz acetate (GA), a United States Food and Drug Administration (FDA)-approved antihypertensive drug, reduces the size and number of nuclear aggregates, improves muscle force, protects myofibers from the pathology-derived turnover and decreases fibrosis [118]. Interestingly, GA increases both the phosphorylation of the eIF2α and sXBP1. It is notable that while the markers of ER stress have been found to be elevated in various muscle disorders, their role in disease progression has not been yet elucidated using pharmacological or genetic approaches.

## 8. Skeletal Muscle Loading

Aerobic and anaerobic exercise leads to beneficial adaptations in skeletal muscle. In addition to promoting skeletal muscle growth as well as metabolic adaptations, exercise inhibits the loss skeletal muscle mass. Interestingly, recent studies have indicated that exercise can induce the activation of all three pathways of the UPR [119,120]. More specifically, a one bout of exhaustive treadmill running increases the expression levels of UPR genes GRP78/BiP, GRP94, GADD34, ATF4, CHOP, and sXBP1 [119]. Furthermore, mice that are run on a downhill treadmill also upregulate ER stress markers phospho-IRE1 (Ser734), phospho-PERK (Thr981), and phospho-eIF2α (Ser52) in the skeletal muscle [121]. However, the literature suggests most adaptations are seen only during acute exercise bouts. Indeed, training the mice for an extended time before sacrifice had a diminished effect on the UPR. Specifically, only the ER chaperones GRP78/BiP and GRP94 were found to be increased in skeletal muscle of trained mice [119]. The effect of acute exercise on ER stress is also demonstrated in rat models. When the rodents were subjected to chronic contractile activity, a similar rapid increase in the markers of the UPR was observed. However, similar to mice, this effect was attenuated with repeated bouts [122]. Lastly, human muscle biopsies after a bout of running have also been shown to increase markers of ER stress [123].

During aerobic exercise, mitochondrial biogenesis is stimulated through the transcriptional coactivator peroxisome proliferator-activated receptor gamma coactivator-1 alpha (PGC-1α) [124]. Interestingly, it was recently discovered that PGC-1α is necessary for the activation of the UPR during pharmacologically induced ER stress and exercise training [119]. Specifically, the cleaved ATF6α (ATF6N) interacts with PGC-1α to stimulate an adaptive UPR in skeletal muscle after aerobic exercise. The importance of ATF6α in aerobic adaptation is further demonstrated by the findings that mice lacking ATF6α have increased inflammation and fail to recover from that muscle damage after acute exercise. Although ATF6α seems to be a part of the adaptive response to acute exercise, other molecules of the UPR may inhibit skeletal muscle adaptation to exercise. Indeed, genetic deletion of CHOP was shown to improve exercise adaptation in skeletal muscle specific PGC-1α knockout mice [119]. Although further investigations are needed, it is plausible that some parts of the UPR aid in adaptation to acute exercise whereas others do not.

Anaerobic exercise occurs when the skeletal muscle is subjected to a short bout of maximal contraction. These exercises cause adaptations to skeletal muscle leading to increases in protein synthesis and organelle biogenesis. A common model used in rodents to induce muscle hypertrophy is the synergistic ablation model [125,126,127,128,129]. In this model, the gastrocnemius and soleus muscles are partially ablated leaving the plantaris muscle as the lone hind limb extensor, mimicking the overload principle used during resistance exercise. Interesting recent data have indicated that a possibly high rate of protein synthesis, which occurs during muscle hypertrophy, results in a maladaptive UPR. More specifically, after synergistic ablation, skeletal muscle increase CHOP and decrease in IRE1α, potentially providing the break to excessive muscle growth through inhibiting protein synthesis [130]. Similar results were also demonstrated in human biopsies after an acute resistance training exercise. The muscle biopsies showed an increase in the protein levels of GRP78/BiP, PERK, and IRE1α 48 h post-exercise. However, the mRNA levels of IRE1α as well as ATF6 where unchanged [131].

Taken together, these studies indicate that acute moderate exercise induces ER stress and activation of UPR pathways. However, after multiple training session, this effect seems to be diminished. This indicated that possibly the UPR only becomes activated to novel or challenging exercises in skeletal muscle during both endurance and resistance exercises. This activation leads to beneficial adaptations, such as increases in mitochondrial capacity. More investigations are needed to better understand the molecular mechanisms of individual molecules of the UPR and what role each has in promoting adaptations to acute exercise.

## 9. Conclusions

ER stress is caused within the cell during numerous pathological conditions. Recent evidence has made it clear that ER stress occurs within skeletal muscle during a number of catabolic conditions including cachexia, denervation, nutrient deprivation, aging, and disease as well as during skeletal muscle loading. Through the use of molecular techniques and genetic manipulations, research has only just begun to elucidate the mechanism by which ER stress and activation of the UPR can lead to skeletal muscle atrophy during these conditions. This review only began to dissect how each one of these specific molecules of the UPR can act independently during these conditions. Further use of genetic manipulations is necessary to provide a complete understanding of how this complex cellular response influences skeletal muscle undergoing catabolic stimuli.

## Abbreviations

%	Percent
4-PBA	4-phenylbutyrate
Aβ	Amyloid-β
AChR	Acetylcholine receptor
ActRIIA	Activin receptor type IIA
ActRIIB	Activin receptor type IIB
4E-BP1/PHAS1	Eukaryotic translation initiation factor 4E (eIF4E)-binding protein 1
AIDS	Acquired immune deficiency syndrome
Akt	Protein kinase B
ALK	Activin receptor-like kinase
ALP	Autophagic-lysosomal pathway
ALS	Amyotrophic lateral sclerosis
ADP	Adenosine diphosphate
AMP	Adenosine monophosphate
AMPK	Adenosine monophosphate (AMP)-activated protein kinase
Apc	Adenomatous polyposis coli
ATF2	Activating transcription factor-2
ATF4	Activating transcription factor-4
ATF6	Activating transcription factor-6
ATF6N	Cleaved N-terminal fragment of activating transcription factor-6 (ATF6)
Atg13	Autophagy-related 13
ATP	Adenosine triphosphate
BiP	Binding immunoglobulin protein
BMP	Bone morphogenic protein
BMPRII	Bone morphogenetic protein receptor type II
Bnip3	Bcl-2 adenovirus E1B 19 kDa-interacting protein 3
C26	Colon-26
Ca^2+^	Calcium ions
CaMK	Calcium/calmodulin-dependent kinase
cIAP1	Cellular inhibitor of apoptosis 1
CHF	Chronic heart failure
CHOP	CCAAT enhancer binding protein (C/EBP) homologous protein (CHOP)
CKD	Chronic kidney disease
CMT	Charcot-Marie-Tooth disease
COPD	Chronic obstructive pulmonary disease
Cu	Copper
DM1	Myotonic dystrophy type 1
DMD	Duchenne muscular dystrophy
DMPK	Dystrophia myotonica protein kinase
DRP1	Dynamin-related protein 1
eIF2	Eukaryotic translation initiation factor 2
eIF2α	Eukaryotic translation initiation factor 2α
eIF2AK3	Eukaryotic translation initiation factor 2 alpha kinase 3
eIF2B	Eukaryotic translation initiation factor 2B
eIF3f	Eukaryotic translation initiation factor 3 subunit f
eIF4E	Eukaryotic translation initiation factor 4E
eIF4G	Eukaryotic translation initiation factor 4G
ER	Endoplasmic reticulum
ERK	Extracellular signal-regulated kinase
ERN1	Endoplasmic reticulum to nucleus signaling 1
Fbox40	F-box protein 40
FDA	Food and Drug Administration
FGF	Fibroblast growth factor
FIP200	Focal adhesion kinase (FAK) family interacting protein of 200 kD
Fn14	Fibroblast growth factor-inducible 14
FoxO	Forkhead box O
GA	Guanabenz acetate
GADD34	Growth arrest and DNA damage-inducible protein 34
GADD45a	Growth arrest and DNA damage-inducible protein 45 alpha
GDF	Growth differentiation factor
GRP78	78 kDa glucose-regulated protein
GRP94	Glucose-regulated protein 94
GSK3β	Glycogen synthase kinase 3 beta
HDAC	Histone deacetylase
HSP90	Heat shock protein 90
IGF-1	Insulin-like growth factor-1
IGF-1R	Insulin-like growth factor-1 receptor
IKK	IκB kinase
IL	Interleukin
IL-1	Interleukin-1
IL-1R	Interleukin-1 receptor
IP_3_R	Type I D-myo-inositol 1,4,5-trisphosphate receptor
IRE	Inositol-requiring protein
IRS1	Insulin receptor substrate 1
JNK	c-Jun N-terminal kinase
LC3	Microtubule-associated protein 1A/1B-light chain 3
LLC	Lewis lung carcinoma
MAFbx/atrogin-1	Muscle atrophy F-box
MAPK	Mitogen-activated protein kinase
mdx	X-linked muscular dystrophy
MG	Myasthenia gravis
MHC-I	Class I major histocompatibility complex
Min	Multiple intestinal neoplasia
MKK4	Mitogen-activated protein kinase kinase 4
MLC	Myosin light chain
MyHC	Myosin heavy chain
mRNA	Messenger RNA
mTOR	Mammalian target of rapamycin
mTORC1	Mammalian target of rapamycin complex 1
mTORC2	Mammalian target of rapamycin complex 2
MuRF1/TRIM63	Muscle RING finger 1
MUSA1	Muscle ubiquitin ligase of the SCF complex in atrophy-1
MyD88	Myeloid differentiation primary response gene 88
Nedd4.1	Neural precursor cell expressed developmentally down-regulated protein 4.1
NFAT	Nuclear factor of activated T cells
NF-κB	Nuclear factor-kappa B
NIK	Nuclear factor-kappa B (NF-κB)-inducing kinase
OPA1	Optic atrophy 1
OPMD	Oculopharyngeal muscular dystrophy
PABPN1	PolyA-binding protein nuclear 1
PGC-1α	Peroxisome proliferator–activated receptor gamma coactivator-1 alpha
PDK1	Phosphatidylinositide-dependent protein kinase 1
PERK	Protein kinase R (PKR)-like endoplasmic reticulum kinase
PIP2	Phosphatidylinositol (4,5)-bisphosphate
PIP3	Phosphatidylinositol-3,4,5-trisphosphate
PI3K	Phosphatidylinositol 3-kinase
PKR	Protein kinase R
PTEN	Phosphatase and tensin homologue
p38	p38 mitogen-activated protein kinase (MAPK)
p70S6K	70-kDa ribosomal protein S6
Rag2	Recombination activating gene 2
REDD1	Regulated development of DNA damage responses 1
Rheb	Ras homolog enriched in brain
RNA	Ribonucleic acid
rpS6	Ribosomal protein S6
SBMA	Spinal and bulbar muscular atrophy
Ser	Serine
shRNA	Short hairpin RNA
sIBM	Sporadic inclusion body myositis
siRNA	Small interfering RNA
SMA	Spinal muscular atrophy
SOD1	Superoxide dismutase 1
SRPK	Serine/arginine-rich protein specific kinase
sXBP1	Spliced XBP1
S1P	Site-1 protease
S2P	Site-2 protease
TAK1	Transforming growth factor beta-activated kinase 1
TβRI	Transforming growth factor beta-receptor type I
TβRII	Transforming growth factor beta-receptor type II
TGFβ	Transforming growth factor beta
Thr	Threonine
TLR	Toll-like receptor
TNF-α	Tumor necrosis factor alpha
TNFR	Tumor necrosis factor receptor
TRAF2	Tumor necrosis factor receptor (TNFR)-associated factor 2
TRAF5	Tumor necrosis factor receptor (TNFR)-associated factor 5
TRAF6	Tumor necrosis factor receptor (TNFR)-associated factor 6
TSC	Tuberous sclerosis complex
TUDCA	Tauroursodeoxycholic acid
TWEAK	Tumor necrosis factor (TNF)-like weak inducer of apoptosis
Ulk	Unc-51 like autophagy activating kinase
UPR	Unfolded protein response
UPS	Ubiquitin-proteasome system
uXBP1	Unspliced XBP1
WFA	Withaferin A
XBP1	X-box-binding protein 1
Zn	Zinc
